# Supplementary material for: Proteasomes of Autophagy-Deficient Cells Exhibit Alterations in Regulatory Proteins and a Marked Reduction in Activity
Source: Cells. 2023 May 30;12(11):1514. doi: 10.3390/cells12111514 (PMC10252828; doi:10.3390/cells12111514)
Supplement: Supplementary file 1 [file cells-12-01514-s001.zip › cells-2353921-supplementary.pdf]

Figure S1

(a)

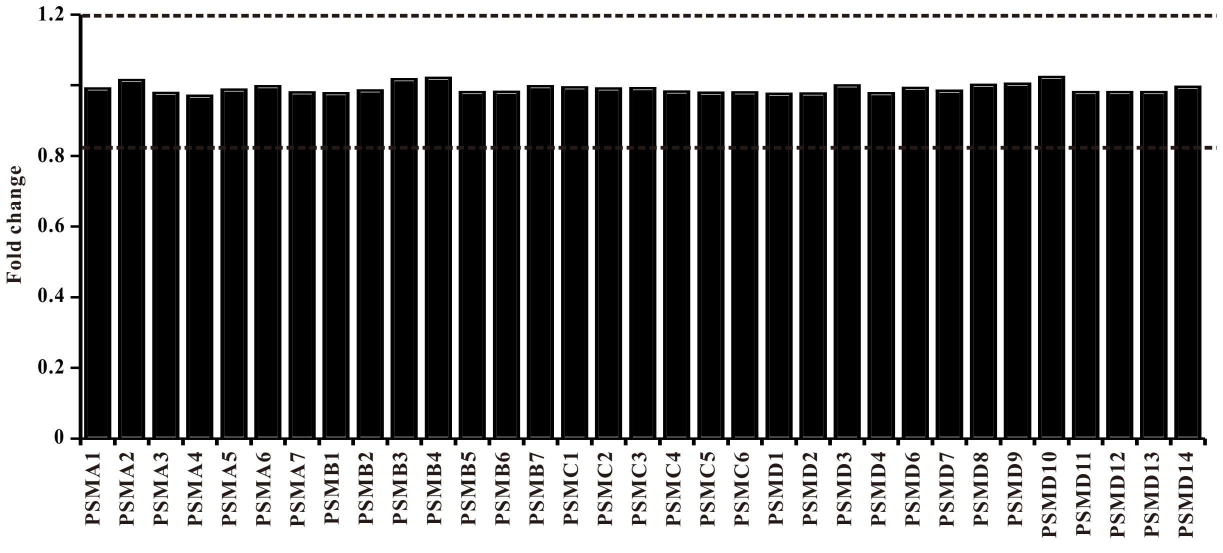

Fold change
1.2
0.8
0.6
0.4
0.2
0
PSMA1
PSMA2
PSMA3
PSMA4
PSMA5
PSMA6
PSMA7
PSMB1
PSMB2
PSMB3
PSMB4
PSMB5
PSMB6
PSMB7
PSMC1
PSMC2
PSMC3
PSMC4
PSMC5
PSMC6
PSMD1
PSMD2
PSMD3
PSMD4
PSMD6
PSMD7
PSMD8
PSMD9
PSMD10
PSMD11
PSMD12
PSMD13
PSMD14

(b)

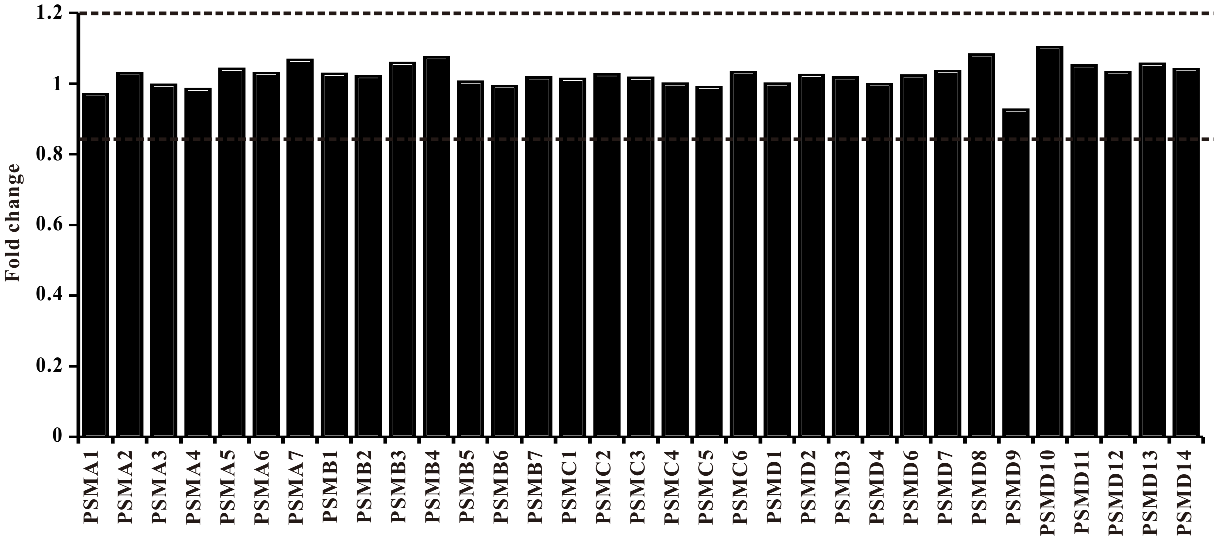

Fold change
1.2
1
0.8
0.6
0.4
0.2
0
PSMA1
PSMA2
PSMA3
PSMA4
PSMA5
PSMA6
PSMA7
PSMB1
PSMB2
PSMB3
PSMB4
PSMB5
PSMB6
PSMB7
PSMC1
PSMC2
PSMC3
PSMC4
PSMC5
PSMC6
PSMD1
PSMD2
PSMD3
PSMD4
PSMD6
PSMD7
PSMD8
PSMD9
PSMD10
PSMD11
PSMD12
PSMD13
PSMD14

Figure S2

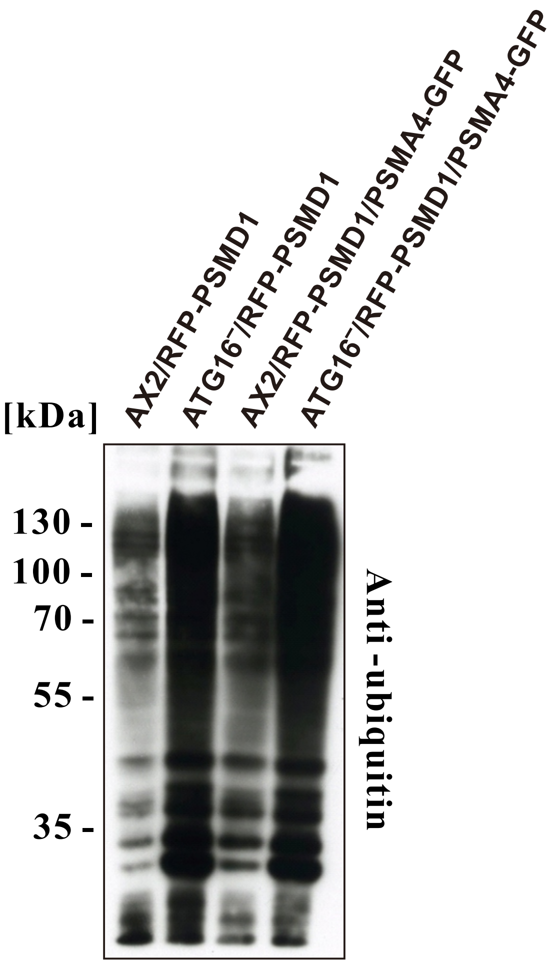
AX2/RFP-PSMD1
ATG16⁻/RFP-PSMD1
AX2/RFP-PSMD1/PSMA4-GFP
ATG16⁻/RFP-PSMD1/PSMA4-GFP
[kDa]
130 -
100 -
70 -
55 -
35 -
Anti-ubiquitin

Figure S3

(a)

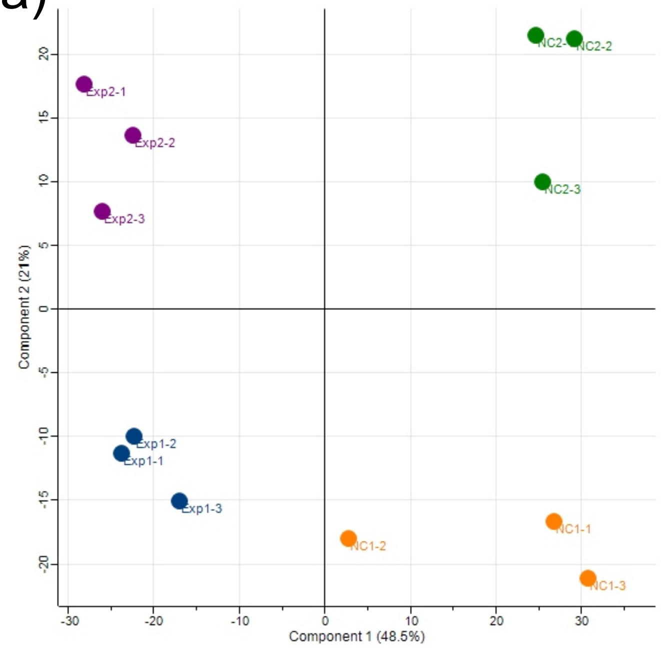

Exp2-1
Exp2-2
Exp2-3
NC2-
NC2-2
NC2-3
Exp1-2
Exp1-1
Exp1-3
NC1-2
NC1-1
NC1-3
Component 2 (21%)
Component 1 (48.5%)
20
15
10
5
0
-5
-10
-15
-20
-30
-20
-10
0
10
20
30

(b)

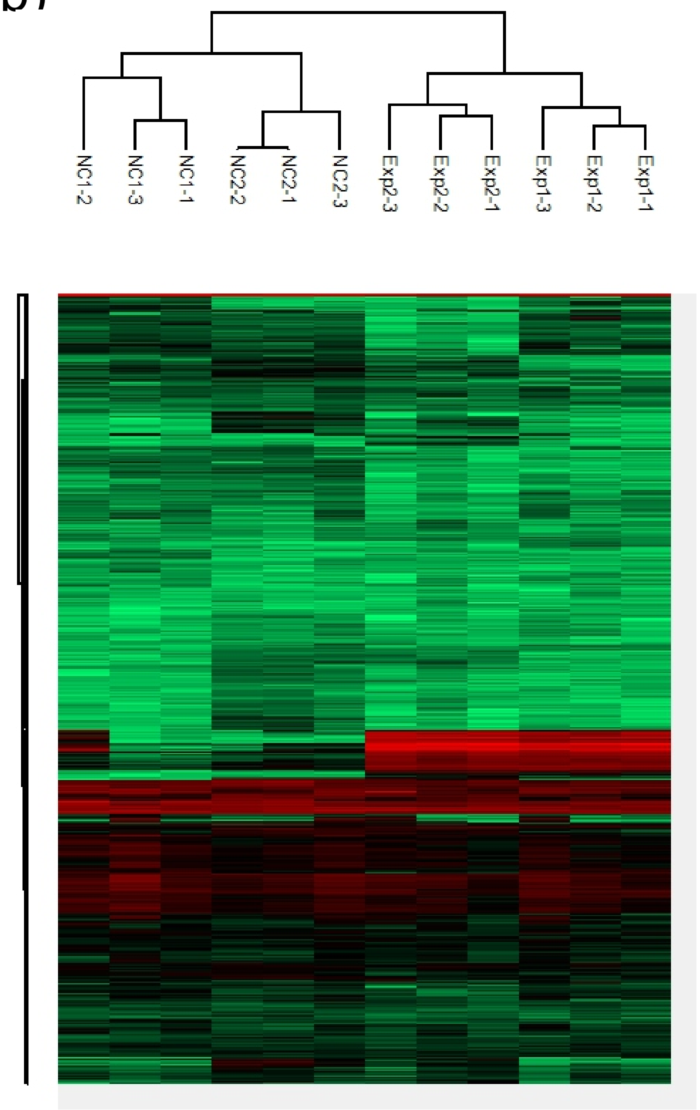

NC1-2
NC1-3
NC1-1
NC2-2
NC2-1
NC2-3
Exp2-3
Exp2-2
Exp2-1
Exp1-3
Exp1-2
Exp1-1

Supplementary Tables

Table S1. Mass spectrometric results of differences of proteasomal subunits and assembly chaperones between the different strains.

	UniProt ID	DDB_G ID	Gene Name	Description	[kDa]	AX2-NC1		ATG16^{-}-NC2		ATG16^{-}-AX2	
						Delta (log2)	p	Delta (log2)	p	Delta (log2)	p
20S proteasome	Q27562	DDB_G0282363	*psmA1*	Proteasome subunit alpha type-1	28.0	7.22	0.024	7.78	0.001	0.56	0.032
	Q54DM7	DDB_G0292122	*psmA2*	Proteasome subunit alpha type-2	25.7	6.06	0.095	5.40	0.011	0.36	0.621
	Q27563	DDB_G0267408	*psmA3*	Proteasome subunit alpha type-3	27.2	7.91	0.031	8.31	0.000	0.70	0.002
	P34119	DDB_G0280969	*psmA4*	Proteasome subunit alpha type-4	28.1	7.31	0.005	7.83	0.003	-0.04	0.928
	Q55G04	DDB_G0268538	*psmA5*	Proteasome subunit alpha type-5	26.7	6.60	0.059	7.80	0.006	0.31	0.192
	Q54XM7	DDB_G0278847	*psmA6*	Proteasome subunit alpha type-6	27.2	7.74	0.016	8.43	0.000	0.69	0.060
	P34120	DDB_G0272831	*psmA7*	Proteasome subunit alpha type-7	27.6	6.81	0.005	6.61	0.000	0.57	0.082
	Q86A21	DDB_G0272969	*psmB1*	Proteasome subunit beta type-1	26.4	6.39	0.060	9.23	0.000	0.62	0.065
	Q55DY7	DDB_G0269472	*psmB2*	Proteasome subunit beta type-2	22.1	6.56	0.041	7.63	0.000	0.46	0.186
	Q55D66	DDB_G0269772	*psmB3*	Proteasome subunit beta type-3	22.8	5.43	0.059	8.69	0.000	0.86	0.212
	Q556Q0	DDB_G0273909	*psmB4*	Proteasome subunit beta type-4	29.0	6.03	0.092	7.47	0.000	0.53	0.230
	Q54BC8	DDB_G0293784	*psmB5*	Proteasome subunit beta type-5	30.3	7.62	0.004	9.05	0.000	0.85	0.065
	Q55GJ6	DDB_G0267390	*psmB6*	Proteasome subunit beta type-6	23.4	6.52	0.106	9.29	0.000	1.04	0.149
	Q54QR2	DDB_G0283679	*psmB7*	Proteasome subunit beta type-7	28.7	8.03	0.008	10.45	0.000	0.99	0.003
Chaperones	Q54WB6	DDB_G0279769	*psmG1*	Proteasome assembly chaperone 1	31.3	6.65	0.125	6.96	0.010	0.33	0.594
	Q869S8	DDB_G0274447	*psmG2*	Proteasome assembly chaperone 2	30.5	7.35	0.033	10.98	0.000	0.48	0.340
	Q55G88	DDB_G0268522	*psmG3*	Proteasome assembly chaperone 3	16.3	3.97	0.243	5.80	0.022	0.85	0.305
	P0C8Z5	DDB_G0304543	*psmG4*	Proteasome assembly chaperone 4	15.5	7.32	0.012	7.70	0.001	0.28	0.680
	Q55G18	DDB_G0267866	*pomp*	Proteasome maturation protein homolog	14.4	5.86	0.000	5.48	0.000	0.61	0.304
Activator	Q967U1	DDB_G0285099	*psmE3*	Proteasome activator 28	26.2	6.92	0.000	7.29	0.000	0.79	0.128
	Q54DB2	DDB_G0292398	*psmE4*	Proteasome activator complex subunit 4	221.1	7.90	0.015	9.44	0.000	0.56	0.162
	Q54JJ7	DDB_G0288007	NA	Proteasome-associated protein ECM29 homolog	212.4	3.67	0.002	3.86	0.002	1.10	0.145

Continued

	UniProt ID	DDB_G ID	Gene Name	Description	[kDa]	AX2-NC1		ATG16^{-}-NC2		ATG16^{-}-AX2	
						Delta (log2)	p	Delta (log2)	p	Delta (log2)	p
19S proteasome (regulatory particle)	Q55BV5	DDB_G0270784	*psmC1*	26S proteasome regulatory subunit 4 homolog	49.2	5.51	0.000	3.99	0.008	0.01	0.978
	Q86JA1	DDB_G0276917	*psmC2*	26S proteasome regulatory subunit 7	47.4	4.34	0.000	3.17	0.000	-0.03	0.762
	Q54PN7	DDB_G0284415	*psmC3*	26S proteasome regulatory subunit 6A homolog	46.9	4.46	0.000	3.11	0.000	-0.19	0.382
	P34123	DDB_G0289003	*psmC4*	26S proteasome regulatory subunit 6B homolog	45.5	4.47	0.000	2.88	0.001	0.03	0.898
	P34124	DDB_G0292382	*psmC5*	26S proteasome regulatory subunit 8	45.2	4.33	0.001	2.90	0.000	-0.15	0.462
	Q54PJ1	DDB_G0284517	*psmC6*	26S proteasome regulatory subunit 10B	44.3	3.91	0.001	3.01	0.000	-0.08	0.680
	Q54JM5	DDB_G0287953	*psmD1*	26S proteasome non-ATPase regulatory subunit 1	107.9	3.36	0.000	3.50	0.000	-0.16	0.344
	Q54BC6	DDB_G0293752	*psmD2*	26S proteasome non-ATPase regulatory subunit 2	98.4	5.42	0.011	5.00	0.000	-0.01	0.979
	Q1ZXD3	DDB_G0288621	*psmD3*	26S proteasome non-ATPase regulatory subunit 3	57.4	6.01	0.000	5.39	0.000	-0.14	0.581
	Q553E0	DDB_G0275775	*psmD4*	26S proteasome non-ATPase regulatory subunit 4	38.4	6.34	0.000	4.72	0.000	0.04	0.935
	Q55C75	DDB_G0270188	*psmD6*	26S proteasome non-ATPase regulatory subunit 6	44.2	5.13	0.000	4.41	0.000	0.20	0.619
	Q54WI8	DDB_G0279633	*psmD7*	26S proteasome non-ATPase regulatory subunit 7	36.3	4.79	0.002	4.19	0.002	-0.02	0.960
	P02889	DDB_G0273979	*psmD8*	26S proteasome non-ATPase regulatory subunit 8	30.4	5.68	0.005	5.15	0.000	-0.01	0.958
	Q54UB5	DDB_G0281315	*psmD11*	26S proteasome non-ATPase regulatory subunit 11	47.6	7.09	0.003	5.38	0.000	0.02	0.929
	Q54UJ0	DDB_G0281051	*psmD12*	26S proteasome non-ATPase regulatory subunit 12	51.6	4.97	0.001	4.49	0.000	-0.03	0.867
	Q54NQ0	DDB_G0285105	*psmD13*	26S proteasome non-ATPase regulatory subunit 13	44.1	8.10	0.000	5.92	0.000	0.17	0.516
	Q86IJ1	DDB_G0272566	*psmD14*	26S proteasome non-ATPase regulatory subunit 14	34.5	4.78	0.008	3.48	0.000	-0.07	0.770
	Q556N5	DDB_G0274093	*adrm1*	Proteasomal ubiquitin receptor ADRM1 homolog	32.3	3.74	0.001	2.24	0.002	1.11	0.048
	Q54K21	DDB_G0287633	*DSS1/ SEM1*	Probable 26S proteasome complex subunit sem1 (DSS1/SEM1 family protein)	9.0	4.43	0.004	4.59	0.000	-0.25	0.692

Three independent experiments were performed with each strain and the precipitated proteins were subjected to mass spectrometry. Differences (delta log2) between the different strains are derived from the calculated mean values from label-free quantification (LFQ). AX2: Wild-type strain expressing RFP-PSMD1 and GFP-PSMA4; NC1: Negative control, i.e. wild-type strain expressing RFP-PSMD1; ATG16^{-}: ATG16^{-} strain expressing RFP-PSMD1 and GFP-PSMA4; NC2: ATG16^{-} strain expressing RFP-PSMD1. NA: not available

Table S2. Proteins exclusively enriched in AX2 or ATG16⁻ cells as highlighted in green.

	UniProt ID	DDB_G ID	Gene Name	Description	[kDa]	AX2-NC1		ATG16⁻-NC2		ATG16⁻-AX2	
						Delta (log2)	p	Delta (log2)	p	Delta (log2)	p
AX2 versus negative control	Q86H60	DDB_G0275525	*hspK*	Small heat shock protein hspK	20.0	2.913	0.004	0.000	1.000	-3.397	0.009
	Q54ST0	DDB_G0282243	*nhp2l1*	NHP2-like protein 1 homolog	14.0	2.775	0.002	-2.059	0.002	-2.365	0.003
	Q54PF3	DDB_G0284597	*csn5*	COP9 signalosome complex subunit 5	37.9	2.679	0.005	-0.921	0.389	-1.495	0.195
	Q55CA0	DDB_G0269168	*vps26*	Vacuolar protein sorting-associated protein 26	40.3	2.306	0.011	0.000	1.000	-2.009	0.007
	Q54E53	DDB_G0291848	*csn3*	COP9 signalosome complex subunit 3	47.3	2.008	0.009	-0.574	0.442	-0.961	0.232
	Q9NIF3	DDB_G0267452	*ubqln*	ubiquilin domain-containing protein	56.7	1.893	0.013	0.766	0.570	-1.172	0.42
	Q1ZXN5	DDB_G0272476	*NA*	putative NADH-ubiquinone oxidoreductase 8B subunit	14.5	1.714	0.010	-2.689	0.002	-1.416	0.014
	Q54WH2	DDB_G0279607	*forA*	profilin binding protein ForA	135.3	1.618	0.015	0.000	1.000	-2.027	0.045
ATG16⁻ v. negative control and ATG16⁻ v. AX2	Q54S82	DDB_G0282617	*psmF1*	proteasome inhibitor PI31 subunit	36.4	0.000	1.000	5.531	0.001	4.272	0.005
	Q54BM8	DDB_G0293552	*NA*	UPF0652 protein	78.3	0.000	1.000	3.875	0.000	3.906	0.036
	Q55BS4	*DDB_G0270388	*NA*	ubiquitin system component Cue domain containing protein	106.9	0.000	1.000	3.784	0.040	3.855	0.052
	Q54N38	DDB_G0285527	*uch2*	Ubiquitin carboxyl-terminal hydrolase isozyme L5	39.3	0.000	1.000	3.165	0.004	3.353	0.001
	Q5FXM5	*DDB_G0270658	*acbA*	Acyl-CoA-binding protein	9.4	-1.493	0.072	2.535	0.022	1.619	0.057
	Q54QY5	DDB_G0283591	*NA*	Uncharacterized protein	32.9	0.000	1.000	1.779	0.004	2.082	0.019
	Q54ET6	*DDB_G0291229	*abpF*	Actin-binding protein F	269.8	0.000	1.000	1.571	0.016	1.858	0.055

Three independent experiments were performed with each strain and the precipitated proteins were subjected to mass spectrometry. Differences (delta log2) between the different strains are derived from the calculated mean values from label-free quantification (LFQ). AX2: Wild-type strain expressing RFP-PSMD1 and GFP-PSMA4; NC1: Wild-type strain expressing RFP-PSMD1; ATG16⁻: ATG16⁻ strain expressing RFP-PSMD1 and GFP-PSMA4; NC2: ATG16⁻ strain expressing RFP-PSMD1. *, the three proteins which were slightly above the threshold p-value of 0.05 in the ATG16⁻ versus AX2 comparison. NA: not available.

Table S3. Exclusively enriched proteasome-associated proteins in the comparison of ATG16⁻ and AX2 cells.

	UniProt ID	DDB_G ID	Gene Name	Description	[kDa]	AX2-NC1		ATG16⁻-NC2		ATG16⁻-AX2	
						Delta (log2)	p	Delta (log2)	p	Delta (log2)	p
1	Q54DL7	DDB_G0292188	DDB_G0292188	von Willebrand factor A domain-containing protein8	83.7	0.00	1.000	-1.69	0.004	5.48	0.000
2	Q54FC2	DDB_G0290969	DDB_G0290969	NA	44.8	-0.80	0.293	-3.15	0.000	4.84	0.000
3	Q54C11	DDB_G0293202	trafH	TNF receptor-associated factor H	49.9	0.00	1.000	-1.16	0.013	4.63	0.001
4	Q75JM6	DDB_G0276057	DDB_G0276057	UBX domain-containing protein 18	60.4	-3.15	0.013	-0.94	0.393	4.43	0.012
5	Q86IB4	DDB_G0275519	NA	NA	34.3	-2.21	0.058	-0.66	0.326	3.85	0.003
6	Q54F93	DDB_G0290997	mppA2	mitochondrial processing peptidase alpha subunit, peptidase M16 family protein	47.7	-0.63	0.127	-2.65	0.005	3.63	0.000
7	Q55BS9	DDB_G0270380	rpl30	S60 ribosomal protein L30	12.1	-3.28	0.001	0.45	0.269	3.48	0.002
8	Q8MPA5	DDB_G0276951	hspG7	heat shock protein Hsp20 domain-containing protein	23.4	0.00	1.000	-0.07	0.926	3.37	0.013
9	Q54B68	DDB_G0293872	idhB	isocitrate dehydrogenase (NAD+) beta subunit	38.7	-0.62	0.336	-1.24	0.121	3.01	0.006
10	Q54P95	DDB_G0284693	DDB_G0284693	cytochrome c oxidase subunit VIb	9.1	0.00	1.000	-1.65	0.125	2.97	0.039
11	Q86AW9	DDB_G0277743	guaD	guanine deaminase	50.7	-2.31	0.017	-1.39	0.017	2.84	0.012
12	Q54GD3	DDB_G0290227	npl4	nuclear protein localization 4	64.4	0.00	1.000	-1.09	0.262	2.83	0.025
13	Q54NX3	DDB_G0284939	DDB_G0284939	thioredoxin domain-containing protein	15.1	-2.68	0.001	-1.25	0.079	2.78	0.007
14	Q76NZ7	DDB_G0277157	nubp2	nucleotide binding protein 2	29.0	-3.65	0.001	-2.25	0.001	2.76	0.005
15	Q54RP2	DDB_G0283003	DDB_G0283003	Iron-sulfur cluster assembly enzyme ISCU, mitochondrial	21.4	-1.25	0.064	-0.76	0.013	2.72	0.000
16	Q54JV0	DDB_G0287859	DDB_G0287859	Zinc finger, N-recognin domain-containing protein	182.5	0.00	1.000	-1.83	0.000	2.69	0.019

17	Q54RZ4	DDB_G0282815	DDB_G0282815	NA	32.5	0.00	1.000	0.31	0.740	2.68	0.021
18	Q54RA2	DDB_G0283293	DDB_G0283293	putative delta-1-pyrroline-5-carboxylate dehydrogenase, mitochondrial	61.6	-3.44	0.012	-1.59	0.024	2.64	0.039
19	P13231	DDB_G0282141	hatA	actin binding protein, hisactophilin I	13.5	-0.01	0.985	-0.57	0.483	2.59	0.022
20	Q556J0	DDB_G0274019	tkt	transketolase	72.0	-0.33	0.525	0.18	0.841	2.56	0.017
21	Q54EW8	DDB_G0291648	lpd	dihydrolipoamide:NAD oxidoreductase	51.8	-1.24	0.026	-1.10	0.025	2.53	0.004
22	Q54DV3	DDB_G0292016	DDB_G0292016	von Willebrand factor A domain-containing protein 5A	102.3	0.00	1.000	2.10	0.088	2.47	0.000
23	Q54VL5	DDB_G0280267	allC	allantoicase	41.8	-0.47	0.366	-1.62	0.113	2.44	0.043
24	Q58A41	DDB_G0289467	DD8-14	AAA ATPase domain-containing protein	75.2	0.00	1.000	-0.43	0.274	2.30	0.030
25	Q75JD5	DDB_G0271678	pckA	Phosphoenolpyruvate carboxykinase (ATP)	62.6	0.00	1.000	-0.42	0.342	2.29	0.003
26	Q54JE4	DDB_G0288127	ogdh	2-oxoglutarate dehydrogenase, mitochondrial	114.1	-2.92	0.017	-1.42	0.130	2.29	0.014
27	Q54KE6	DDB_G0287377	mccA	Methylcrotonoyl-CoA carboxylase subunit alpha, mitochondrial	77.1	-1.76	0.069	-1.17	0.029	2.29	0.016
28	Q556G1	DDB_G0272628	DDB_G0272628	zinc-containing alcohol dehydrogenase (ADH)	36.8	-0.69	0.169	-0.66	0.021	2.27	0.002
29	Q86KZ6	DDB_G0272322	DDB_G0272322	amidinotransferase	36.2	-0.34	0.726	-3.46	0.021	2.27	0.049
30	Q869S7	DDB_G0274449	scsB	Succinate--CoA ligase [GDP-forming] subunit beta, mitochondrial	45.7	0.00	1.000	-0.35	0.264	2.25	0.002
31	Q557J6	DDB_G0273517	abpE	Drebrin-like protein	55.3	-1.09	0.266	-0.15	0.623	2.18	0.013
32	Q54X73	DDB_G0279159	aco1	Probable cytoplasmic aconitate hydratase	98.0	-2.44	0.013	-1.39	0.054	2.10	0.019
33	Q8T137	DDB_G0272754	gsr	Glutathione reductase	50.5	-0.73	0.209	0.51	0.494	1.93	0.031
34	Q9U1M8	DDB_G0274455	myoI	Myosin-I heavy chain	268.3	0.00	1.000	0.12	0.788	1.90	0.013
35	Q54SU8	DDB_G0282209	DDB_G0282209	Uncharacterized protein	77.1	0.00	1.000	0.04	0.902	1.79	0.003
36	O15692	DDB_G0285251	rsc43	Anamorsin homolog	28.0	-0.83	0.017	-0.09	0.897	1.75	0.048
37	Q54FV6	DDB_G0290575	DDB_G0290575	Uncharacterized protein	53.3	-0.18	0.711	-0.45	0.251	1.74	0.007

38	Q54EW2	DDB_G0291301	DDB_G0291301	Putative bifunctional amine oxidase DDB_G0291301	120.7	0.68	0.198	0.56	0.220	1.70	0.034
39	Q556G3	DDB_G0272632	gsta2-1	Putative glutathione S-transferase alpha-2	22.5	0.00	1.000	-0.02	0.981	1.69	0.047
40	Q8I0H7	DDB_G0293298	mhsp70	Heat shock 70 kDa protein, mitochondrial	71.4	-0.59	0.076	-0.41	0.362	1.65	0.002

Three independent experiments were performed with each strain and the precipitated proteins were subjected to mass spectrometry. Differences (delta log2) between the different strains are derived from the calculated mean values from label-free quantification (LFQ). AX2: Wild-type strain expressing RFP-PSMD1 and GFP-PSMA4; NC1: Wild-type strain expressing RFP-PSMD1; ATG16$^-$: ATG16$^-$ strain expressing RFP-PSMD1 and GFP-PSMA4; NC2: ATG16$^-$ strain expressing RFP-PSMD1. Light orange background, proteins decreased in the ATG16$^-$ versus NC2 comparison but enriched in the ATG16$^-$ versus AX2 comparison; Light blue background, proteins enriched in the ATG16$^-$ versus NC2 and in the ATG16$^-$ versus AX2 comparison, all with p-values above 0.05 in the ATG16$^-$ versus NC2 comparison.
